# Novel concept-based image captioning models using LSTM and multi-encoder transformer architecture

**DOI:** 10.1038/s41598-024-69664-1

**Published:** 2024-09-05

**Authors:** Asmaa A. E. Osman, Mohamed A. Wahby Shalaby, Mona M. Soliman, Khaled M. Elsayed

**Affiliations:** 1 Information Technology Department, Faculty of Computers and Artificial Intelligence, Cairo University, Giza, Egypt; 2 Smart Engineering systems research center (SESC), Nile University, Giza, Egypt; 3 School of Computational Science and Artificial Intelligence, Zewail City, Giza, Egypt

**Keywords:** Image Captioning, Transformer, Computer Vision, Concept Modeling, Natural Language Processing, Information technology, Computer science

## Abstract

Captioning an image involves using a combination of vision and language models to describe the image in an expressive and concise sentence. Successful captioning task requires extracting as much information as possible from the corresponding image. One of these key pieces of information is the topic to which the image belongs. The state-of-the-art methods used topic modeling depending only on caption text in order to extract these topics. The problem with extracting the topics using topic modeling only on caption text is that it lacks the consideration of the image’s semantic information. Instead, concept modeling extracts the concepts directly from the images in addition to considering the corresponding caption text. Concept modeling can be used in image captioning to extremely capture the image contexts and benefit from it to produce more accurate descriptions. In this paper, novel image captioning models are proposed by utilizing the concept modeling technique. The first concept-based model is proposed by utilizing LSTM as a decoder while the second model is proposed in association with new multi-encoder transformer architecture. Standard metrics have been used to evaluate the proposed models using Microsoft COCO and Flickr30K datasets. The proposed models outperformed the related work methods with reduced computational complexity.

## Introduction

The image captioning task involves producing a plausible sentence to describe an image. Image captioning is a needed task, since it is essential to many applications. Over the past two decades, image captioning has been used often in medical area, robotics, information retrieval, and image indexing^1^. To produce a plausible description for an image, image captioning task includes two major models namely, feature extraction and sentence generation^2^. The extraction of the needed features is done based on computer vision approaches. On the other hand, the generation of the sentence (caption) is produced using Natural Language Processing (NLP) models^2^.

To achieve higher accuracy of image captioning complex task, deep learning techniques were extensively employed in the task of captioning images^3^. Based on the literature^3^ deep learning techniques, such as retrieval-based techniques, template-based techniques, end-to-end learning, which includes attention-based techniques, have been used with successful results. In image captioning task, attention-based techniques represent now the state-of-the-art^4^. The attention techniques were first employed in the machine translation task^5^. Based upon significant enhancement of the results in machine translation applications, the attention techniques were then employed in image captioning application^6^. The attention-based technique concentrates on the prominent image regions and the effective features, then it utilizes these data to select where to focus next while generating the corresponding caption. Different attention-based approaches were developed for the purpose of enhancing the resulting captions. As mentioned in^4^, the attention-based techniques may be classified depending on the visual attention part, the blocks included in the attention, the number of layers needed to apply the attention, or finally, the attention technique can be considered as guided-attention.

As indicated in^4^, guided attention technique is more adequate to be used in image captioning due to its considerable improvement in the results. Image captioning process can be guided using information produced from the image features or using text-based information. Topic modeling is commonly used to guide the captioning process^7,8^. Topic statistical modeling is widely used to extract the latent variables from massive datasets. Topic modeling technique was originally developed for textual data. In image captioning, topic modeling is applied to the captions text then modeled on the images in a supervised way^7,8^. However, the extracted topics cannot successfully reflect the image contexts, since they lack the consideration of the image semantic information. Therefore, it is needed to find a suitable approach by which the topics can be extracted from the images directly associated with the ground truth captions. For this purpose, M. Grootendorst proposed the concept modeling technique by customizing the topic modeling to consider images (Maarten Grootendorst, https://github.com/MaartenGr/Concept).

Couple of years ago, concept modeling technique was developed as a novel multimodal technique that uses the images and their annotated captions to extract the corresponding concept vectors. Concept vector is formulated to successfully capture the image’s semantic information. Each concept vector, corresponding to an image, has a length equal to number of the concepts. This vector represents the concepts distribution for the corresponding image. The concept modeling technique is implemented on the images and their corresponding texts using the Contrastive Language-Image Pre-Training (CLIP)^9^ model and BERTopic^10^ model.

As mentioned earlier, the image captioning task is challenging because it necessitates a deep comprehension of the image’s semantic features, and hence the image captioning model should be able to generate human-like descriptions. In this research paper, novel concept-based models are proposed for image captioning. First, a concept modeling technique is employed to extract the image’s concept vector. The resulting concept vector and the CLIP image’s embedding are fed to a decoder block, which generates the image captions. For topic-based image captioning models, LSTM^11^ has been traditionally used to decode the embeddings of the partial captions and the topic vectors to generate the subsequent word of the caption. Hence, concept-based model for image captioning using LSTM is then proposed. In the proposed concept-based model using LSTM, the concept vectors are fed to the LSTM as initial states. Then, LSTM is implemented on the embeddings of the partial captions. The LSTM output is then merged with the CLIP image’s embedded features to predict the next caption words. Since the concept modeling technique is more comprehensive than the topic modeling and it can extremely capture the image contexts. So, the proposed concept-based model using LSTM should be capable of enhancing the results of the state-of-the-art topic-based methods.

The well-known machine translation technique, i.e., transformer, is an attention-based deep learning technique. As indicated in^4^, transformer is used in image captioning task for producing more expressive captions at lower computational complexity because of its parallelization architecture and the recurrence bypassing. However, the traditional transformer has only one encoder in order to encode the image features and one decoder in order to decode the partial captions by attending to the encoded features. As indicated in our first proposed concept-based model for image captioning using LSTM, the decoder module has two inputs which are concept vectors and CLIP image’s embeddings. Therefore, in this paper, a concept-based model using a new multi-encoder transformer architecture is proposed for image captioning. The new proposed multi-encoder transformer architecture is capable of handling the concept vectors and CLIP image’s embeddings for the purpose of attending to the concept information. It is proposed to modify the architecture of the traditional transformer by adding a new encoder for encoding the concept vectors. Further, an encoder-decoder attention layer is added in the decoder for attending to the concept information encoded by the new encoder. In the proposed concept-based model using multi-encoder transformer architecture, the concept modeling technique is used to extract the concept vectors and the CLIP image’s embeddings. After that, the CLIP image’s embeddings are fed to the first encoder and the concept vectors are fed to the new added encoder. Finally, the output of the two encoders in addition to the embeddings of the partial captions are passed to the transformer decoder for predicting the subsequent caption words. Hence, the proposed concept-based model using multi-encoder transformer architecture is capable of enhancing the accuracy of achieved image captions through combining the capabilities of the concept modeling technique and the attention-based transformer technique.

The proposed concept-based models for image captioning are evaluated on Microsoft COCO (MSCOCO) dataset^12^ and Flickr30K^13^ using BLEU^14^, CIDEr^15^, ROUGE^16^, METEOR^17^, and SPICE^18^ metrics.

This paper includes the following contributions:

- This paper proposes novel concept-based models for captioning images. This research work aims to explore the use of the novel image concept vector in the captioning process, towards improving the predicted image caption, since the concept vectors have been formulated to capture the image context efficiently.
- First, a novel concept-based model is proposed for image captioning by utilizing LSTM. Such that the concept modeling technique generates concept vectors that reflects the image’s semantic information which are then fed to an LSTM as initial states. LSTM is then employed on the embeddings of the partial captions and then merged with the images features to produce the image corresponding caption.
- Then, a novel concept-based model is proposed for image captioning in association with a new multi-encoder transformer architecture (META). Such that the new multi-encoder transformer architecture is capable of handling both the CLIP feature vectors and the concept vectors simultaneously for the purpose of representing the concept information.
- The proposed multi-encoder transformer architecture (META) is designed by utilizing multiple encoders for different feature vectors. And hence, a new encoder-decoder attention layer is added in the decoder block for each new added encoder. For the proposed concept-based model using multi-encoder transformer architecture, two encoders are utilized, the first encoder is utilized for the image features and the second one for the concept vectors.

This paper is structured as follows: Sect. “Related work” includes the related works; the proposed models are illustrated in Sect. “Proposed concept-based models for image captioning”; the experimental details in addition to the results are included in Sects. “Experimental work and results” and “Conclusion and future work” includes the conclusion of the paper.

## Related work

Since the development of several deep neural networks, research into image captioning task has been steadily growing. The effectiveness of the models has grown over time, from retrieval-based and template techniques to encoder-decoder techniques including attention-based techniques. Attention-based techniques have recently emerged as the most effective methods in image captioning field^4^. Several attention-based approaches were proposed with the intention of improving the captions accuracy. According to^4^, the attention-based techniques can be broken down into subcategories based on either the visual attention part, the blocks included in the attention, the number of layers needed to apply the attention, or finally, the attention technique can be considered guided-attention.

### Topic-based image captioning related work

**Figure 1:**
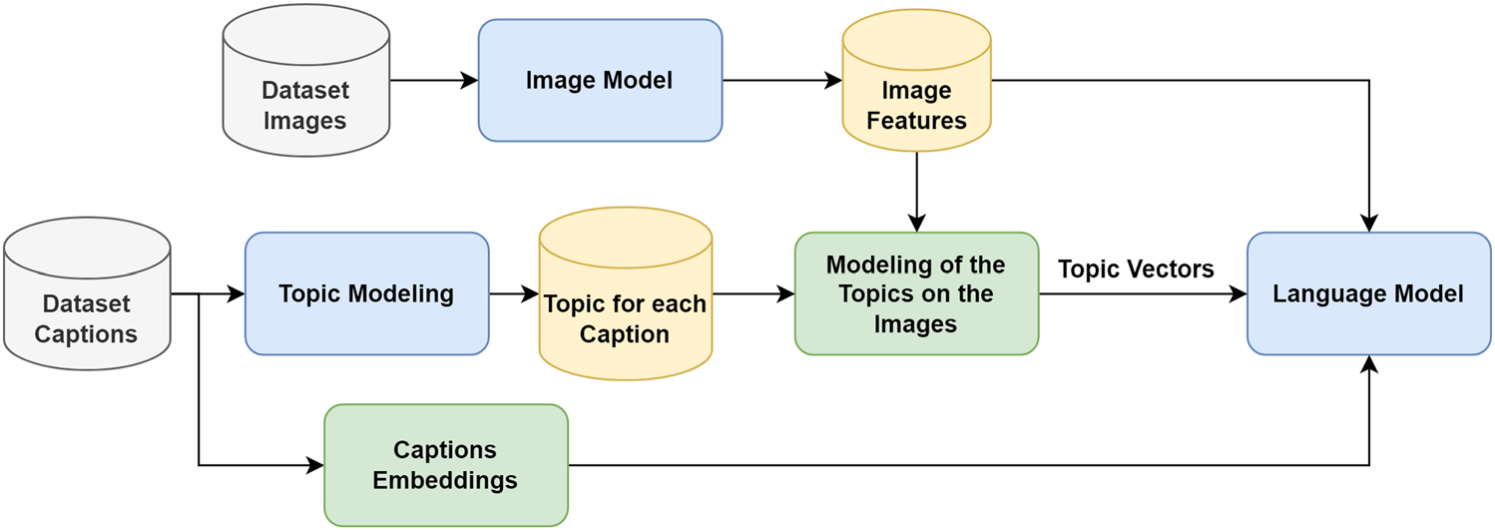
General framework of the topic-based image captioning techniques.

The image captioning process may be guided either using text-based information or using information derived from the image features. Topic modeling is commonly used to guide the captioning process^7,8^. Topic modeling is one of the data analysis tools, and it is more suitable for text documents to define the topic discussed in the document^19^. Figure 1 presents a general framework of the topic-based image captioning techniques. As presented in the figure, the three main modules of the topic-based image captioning methods are the image model, topic modeling and language model. The image model is used for extracting the image features while the topic modeling technique is used to extract the corresponding topics. After that, the language model is utilized for producing the captions using the image features, the topic vectors and the partial captions embeddings. As long as the topic modeling is a text-based technique and it is implemented only on the caption texts, so a modeling is needed to train the images on the extracted topics in a supervised way^7,8^.

Dash et al.^7^ proposed topic-based captioning by employing Latent Dirichlet Allocation (LDA)^20^ to the caption texts to determine the image topics, and then the topics were used as input to guide the captioning process. Inception v3^21^ was utilized in the topic extraction after using LDA, but a fully connected layer had been utilized in association with a sigmoid function to replace the layer of prediction. The extracted topics were merged with Inception v3 features and the texts embedding in the decoding layer to predict the new caption words. Mao et al.^22^ utilized LDA algorithm for topic extraction, and then the extracted topics were incorporated into the caption generation process with a Fusion Gated Unit as guidance for the generated caption to a specific topic. In^23^, the topic information extracted by LDA was integrated into a CNN-RNN model for sentence generation.

Since attention-based approaches are now state-of-the-art^4^, topic modeling was used to guide the attention process in generating the image captions. In^8^, LDA was first applied to the caption texts, then a VGGNet classifier with one label was trained on the image and the extracted topic pairs. Afterwards, the topic as a guidance vector was passed to the attention module in addition to the extracted features for generating the corresponding caption. Popattia et al.^24^ used LDA to extract image topics, and the topics were then predicted using a multi-label classifier. Afterwards, the extracted topics, the image features, and the captions were embedded using a retrieval module, and finally, two LSTMs were used as a decoder. LDA was also used in^25^ for topic extraction, and then a topic-scene graph was proposed to make use of the image topics in a scene graph model.

However LDA is a popular technique for topic extraction, Zia et al.^26^ used the Hierarchical Dirichlet Process (HDP)^27^ for topic extraction and assignment of topics to vocabulary words. Their captioning algorithm uses two CNNs to extract two different features: the scene-based general features in addition to region-based detailed features. Then, their approach used bidirectional LSTM for generating caption words. Yu et al.^28^ used non-negative matrix factorization (NMF)^29^ besides the LDA; a term frequency vector was utilized for the LDA, and a term frequency-inverse document frequency vector was utilized to the NMF. Hierarchy of the topics, ground truth captions, and images is used, with the captions in the middle and the image at the top, and then an order-embedding procedure is used to embed them into one space. Zia et al.^30^ proposed a topic sensitive approach for image captioning. Their approach produces descriptions for the target images by capturing the semantic relationship and polysemous characteristics of the words used to describe the images.

Table 1 presents a comparison of the topic-based approaches in terms of the used topic, image, and language models. It is clear in Table 1 that the topic-based methods in image captioning extract the topics using text-based modeling from the captions, and then train on the images as image,topic pairs in a supervised way. To handle the issue of the topic modeling that considers only text like and ignores the images semantic information, a concept-based approach is proposed for extracting the image concepts from the images and their corresponding ground truth captions.

**Table 1:** Summary of the used topic, image, and language models in the topic-based approaches.

	Topic modeling technique	Image encoder	Language model
Topic-based captioning^7^	LDA	Inception-V3	LSTM
Show and tell more^22^	LDA	ResNet-152	Two layers of LSTM
What topics do images say^23^	LDA	VGGNet	LSTM
Topic-oriented (NeuralTalk2-T-oe)^28^	LDA & NMF	VGGNet	LSTM
Topic-guided attention (VA)^8^	LDA	VGGNet	LSTM with attention
Topic-sensitive^30^	HDP	AlexNet	LSTM

### Transformer-based image captioning related work

Transformer had been introduced in image captioning by Zhu et al.^31^. Zhu et al.^31^ utilized CNN as the image encoder and used a transformer decoder in order to produce the sentence as captioning decoder. Gated Bilateral Controller (GBC) and EnTangled Attention (ETA) were introduced to supplement the traditional transformer by Li et al.^32^. ETA enabled the change to make use of both semantic notions and visual data. The GBC managed the flow of data among the various channels. It was presented by Herdade et al.^33^ that the object relation transformer used spatial attention to incorporate geometrical data related to the relationship among each set of items. He et al.^34^ put up a concept that proposes modifying the transformer’s internal structure. In order to deal with parent-child-neighbor dynamics, they devised a new, more complex transformer with three simultaneous sub-transformer levels. In order to simulate object-to-object, word-to-object, and word-to-word relationships, Yu et al.^35^ suggested a Multimodal transformer. Co-attention in several modalities as well as self-attention in a single modality were learned. Also, there were two different ways to use multiple views: unaligned and aligned multi-views. A transformer approach was put up by Cornia et al.^36^ to take into account both high-level and low-level interactions through a multi-level modeling. When encoding the connections to previous information, they used persistent memory vectors. Additionally, all of the encoder levels contributed to sentence production and were linked to the layers of the decoder in a mesh-like manner rather than focusing attention just on the final encoding layer.

As presented in Table 1, all the included topic-based approaches used LSTM as a language model, and transformer was not utilized. So, transformer was used in the proposed approach of this paper as a language model due to its ability to produce better results when compared to the other attention-based approaches^4^. In the proposed work, the transformer architecture has been modified by adding a new encoder in order to be guided using the concept information and an encoder-decoder attention layer is added to the decoder. Inspired by the approach of Mokady et al.^37^ that used CLIP embeddings for complexity reduction. In the proposed work, the CLIP features, extracted while generating the concepts in the concept modeling technique, have been used in order to enhance the predicted caption and reduce the computational cost of the proposed model.

A multi-encoder transformer network was proposed by Shin and Lee^38^ for a text-based task, i.e., automatic post-editing. The authors updated the transformer to have two encoders: one encoder exists for the output of the machine translation while the second encoder exists for the source statement. In addition, they updated the transformer decoder to have three encoder-decoder attention (Enc-Dec-Atten) layers. The first Enc-Dec-Atten layer represents the dependency between the source statement and the ideal translation. The second Enc-Dec-Atten layer represents the dependency between the source statement and the output of the machine translation. The third Enc-Dec-Atten layer represents the dependency between the previous two Enc-Dec-Atten layers so that the decoder is assisted by the source context in identifying the common terms that are required to be kept in the phrase after editing. The multi-encoder transformer network has been used in text-based applications including text summarization^39^ and machine translation^40,41^. Compared to the multi-encoder transformer architecture of^38^ which is designed for a text-based task, in this paper, new multi-encoder transformer architecture (META) is proposed which is suitable for image captioning task. Two encoders are included in the proposed multi-encoder transformer architecture (META) for two different input feature vectors and the decoder includes two Enc-Dec-Atten layers for the dependency between the encoders and the captions embeddings.

## Proposed concept-based models for image captioning

**Figure 2:**
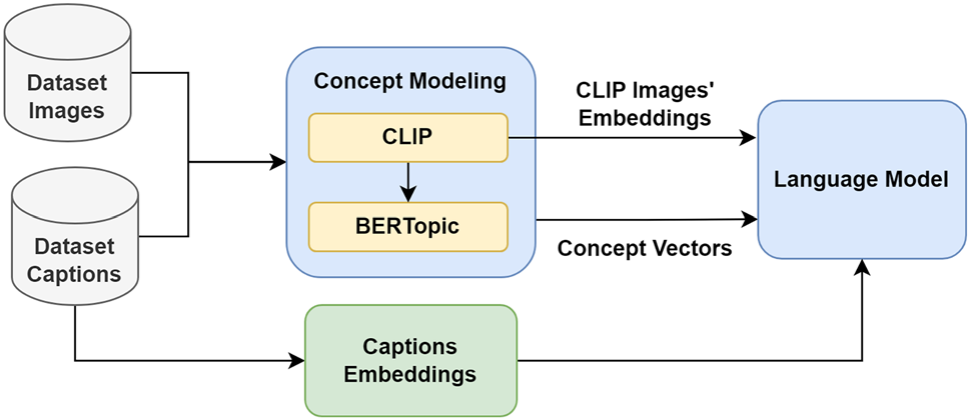
General framework of the proposed concept-based image captioning models.

In this research work, novel concept-based models are proposed for image captioning. First, concept modeling technique is implemented to extract the image’s concept vector. The output concept vector and the CLIP image’s features are fed to a language model, which produces the image captions. The general framework of the proposed models is presented in Fig. 2. By comparing Fig. 2 with Fig. 1, there is no need to have an image model because the CLIP model is already involved in the concept modeling technique. So, the CLIP images’ embeddings are used in this research work as the image feature vectors. In addition, there is no need to have additional module for modeling the concepts on the images like the topic-based models because the images are already involved in the concepts’ extraction process. In the first proposed concept-based model, an LSTM is utilized as the language model. However, in the second proposed concept-based model, a new proposed multi-encoder transformer is used as the language model.

### Concept modeling technique

Concept modeling technique has been developed two years ago by M. Grootendorst through customizing the topic modeling to consider images. Concept modeling technique is a multimodal technique that generates set of concept vectors based on the input images and their corresponding texts using CLIP^9^ model and BERTopic^10^ model. The principle behind the concept modeling technique is to extremely capture the image’s semantic information.

CLIP is a multimodal paradigm that combines understanding of English-language topics with understanding of the semantics of the image. Instead of separately having an image encoder for extracting images feature vectors and a text classifier to produce label predictions, CLIP^9^ can jointly train both encoders. It was trained over a broad set of 4 million image and text pairs. For each batch of the training dataset, CLIP learns the correlated representations between the images and texts by maximizing the similarity, specifically cosine similarity, between the accurate image,text embeddings and lowering the similarity of the mismatched pairs.

BERTopic is a topic modeling technique that relies on embeddings derived from BERT pre-trained approaches^10^. BERTopic considers the process of turning texts into topic-words through three main modules. The documents are first transformed into embeddings, after that their dimensionality is reduced, they are clustered, and then ultimately transformed into topics.

**Figure 3:**
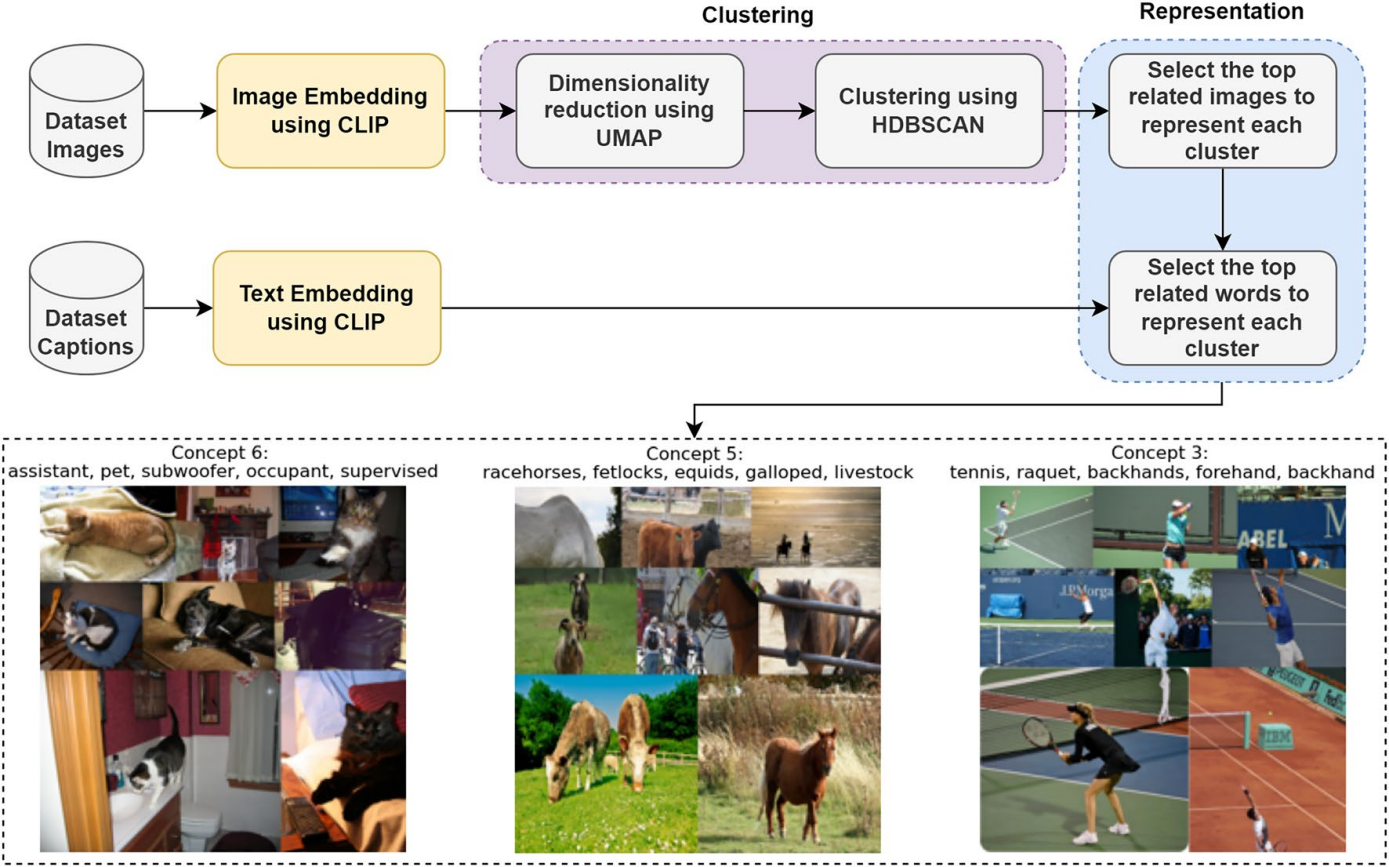
Concept modeling technique main steps.

The concept modeling technique presented in Fig. 3, starts by using the CLIP model in order to embed the input images and the corresponding captions into the same vector space. Then, the acquired embeddings must be subjected to clustering. Numerous clustering techniques are sensitive to a dimensionality issue. The dimensionality issue basically means that it becomes challenging to cluster the points when they are dispersed across numerous dimensions, because most of the points end up being distant from one another. So, the dimensionality of the embedding vector for every sentence must first be reduced to lower dimensions. As a result, Uniform Manifold Approximation and Projection (UMAP)^42^ has been used for reducing the dimensions in order to efficiently cluster the data. Hierarchical Density-based Spatial Clustering of Applications with Noise (HDBSCAN)^43^ was then used for clustering the embeddings of the images into a set of clusters having common semantics. HDBSCAN is a density-based method that groups the points into clusters according to how easily they can be reached. The points that are distant from the majority of the other points are considered outliers. After that, the concepts can be represented by choosing the relevant images for each cluster based on Maximal Marginal Relevance (MMR). The final step is using cosine similarity to select the keywords that represent each concept cluster.

### Proposed concept-based model using LSTM (CM-LSTM)

As explained earlier, the proposed concept-based models start with employing the concept modeling technique to extract the concept vectors and the CLIP features. Then, a language model is used on the output of the concept modeling technique and the partial captions embeddings. In this first proposed model, an LSTM is used as a language model for decoding the partial embeddings and the concept modeling output to produce the subsequent caption word.

In the proposed model, images with the ground truth captions are fed as input to the concept modeling technique. The concept modeling technique starts by using the CLIP model for embedding the images and their captions. After that, the embedding dimensions are reduced using UMAP and then clustered using HDBSCAN. The main output of the concept modeling technique is the concept vectors. Each concept vector, corresponding to an image, has a length equal to number of the concepts. This vector represents the concepts distribution for the corresponding image. In order not to have additional image encoder like the topic-based models, the CLIP images’ embeddings, which are produced within the process of extracting the concepts vectors in the concept modeling technique, are employed in this work as the images’ feature vectors.

In the proposed concept-based model using LSTM (CM-LSTM), the extracted concept vectors were fed to an LSTM as the initial states. Then, LSTM is applied to the embedded partial captions and then merged with the CLIP embedded features for producing the caption words. A Pseudo-code Algorithm of the proposed concept-based model using LSTM is presented in Table 2.

**Table 2:**
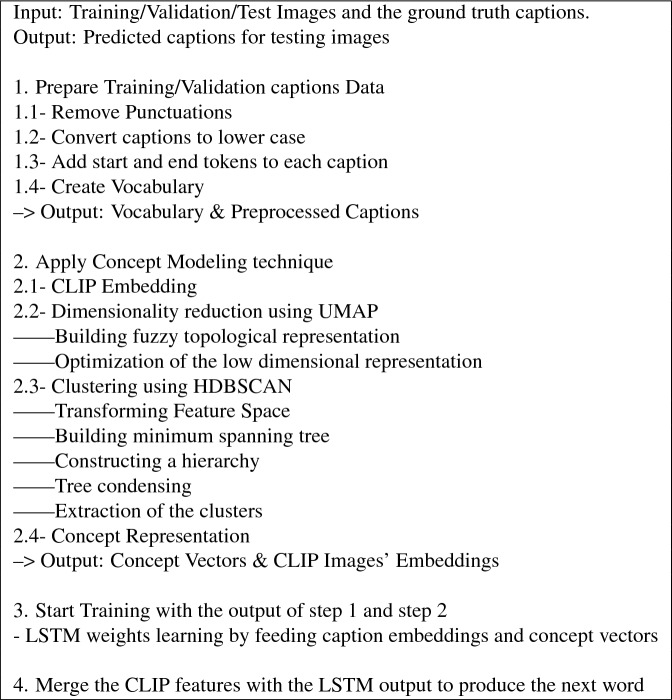
Algorithm of the proposed concept-based model using LSTM (CM-LSTM).

### Proposed multi-encoder transformer architecture (META)

For the purpose of producing more expressive captions at lower computational complexity, transformer is used in the second proposed concept-based image captioning model because of its parallelization architecture and the recurrence bypassing. Therefore, it is proposed to modify the traditional transformer by having multi-encoder architecture for representing the novel concept vectors in the captioning process.

Transformer is an attention-based architecture for boosting the quality of deep learning text translation systems. It was first presented by Vaswani et al.^44^, and it has since become the standard framework for the majority of language models. The traditional transformer architecture includes only one encoder and one decoder each with several identical layers. The encoder block includes self-attention module in addition to fully connected feed-forward network (FF-Network)^44^.

Self-attention module is a multi-head attention where the output of each encoder layer is used to provide the keys, values, and queries for the subsequent layers. Each place in the encoder has the ability for attending to all of the positions at the encoder’s preceding layer. Multiple levels of scaled attention constitute the multi-head attention (MHA) layer. The scaled attention over the queries $Q$, keys $K$, and values $V$ can be calculated as follow^43^:

1 $$\begin{aligned} Att(Q,K,V)=softmax((QK^T)/\sqrt{d})V \end{aligned}$$

where $d$ is the dimensionality shared by all input features. The queries $Q$, keys $K$, and values $V$ are linearly projected and then submitted to scaled attention in every head. Afterwards, multi-head attention output ($MHA$) is made by concatenating the outputs of all $D$ heads and then projecting them.

2 $$\begin{aligned} h_j= & {} Att(QW_j^Q,KW_j^K,VW_j^V) \end{aligned}$$

3 $$\begin{aligned} MHA= & {} Concatenate(h_1,..,h_j,..,h_D ) W^o \end{aligned}$$

where $W_j^Q, W_j^K, W_j^V$ are the matrices used for projection of the $j$-th head, and $W^o$ is the matrix used for output projection of the heads. A feed-forward layer comes after the self-attention module, that is composed of the ReLU activation function and dropout between two linear layers, as follows:

4 $$\begin{aligned} FF(x)=Linear(Dropout(ReLU(Linear(x)))) \end{aligned}$$

The decoder block is composed of the same modules as the encoder in addition to an encoder-decoder attention (Enc-Dec-Atten) layer that attends to the encoder output. In the encoder-decoder attention layer, the output of the prior decoder layer is used to provide the queries, whereas the encoder output is used to provide the memory values and keys. This layer gives the opportunity to each place within the decoder for attending to each and every position within the input sequence.

**Figure 4:**
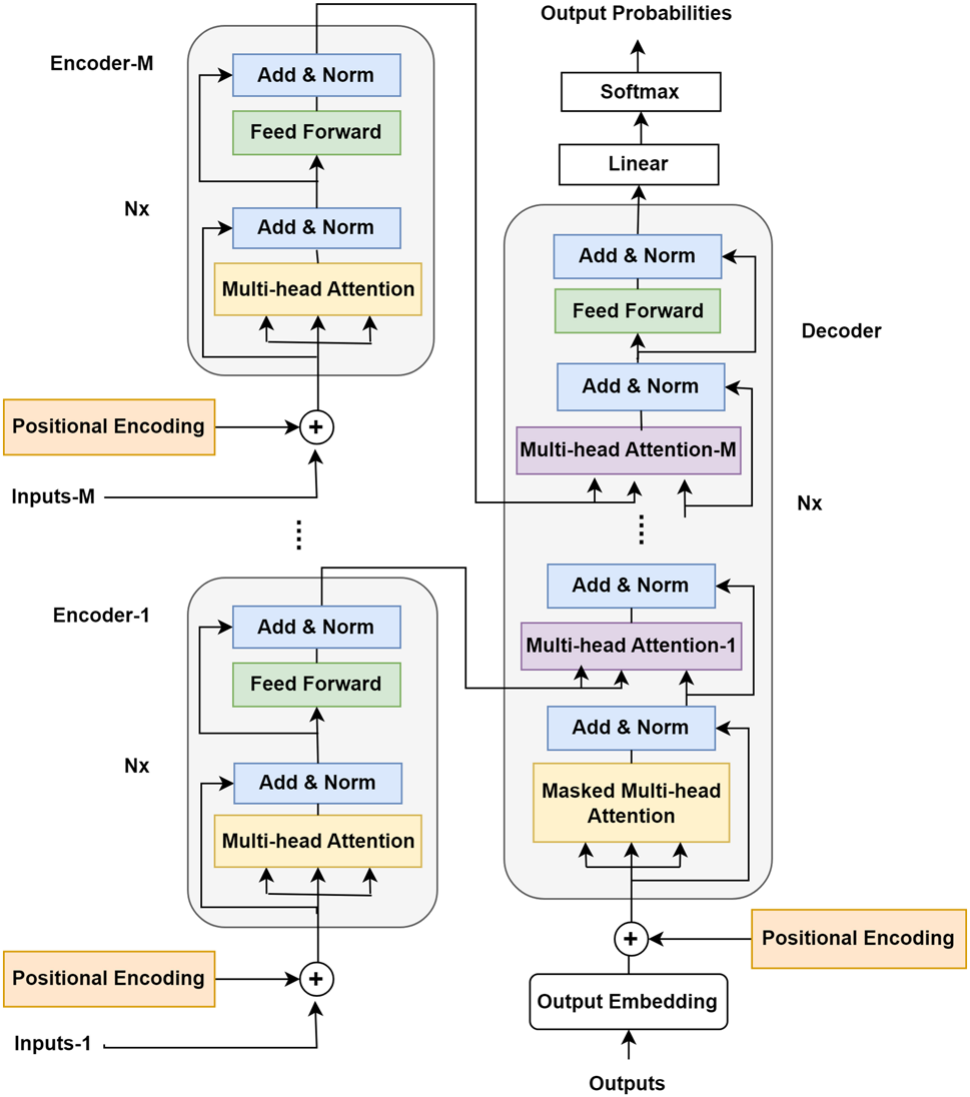
The proposed multi-encoder transformer (META) architecture includes M encoders and one decoder.

As mentioned above, the traditional transformer has only one encoder which handles one feature vector and one decoder. On the other hand, there are some situations in which there is a need to apply attention on more than one feature vector simultaneously. Such as the problem is being considered in this paper, the image captioning, there is a need to use several feature vectors representing different information related to the images. A multi-encoder transformer architecture (META) is proposed by adding multiple encoders to deal with multiple input of feature vectors and hence encoder-decoder attention (Enc-Dec-Atten) layers will be added to the decoder for each encoder. The generic architecture of the new proposed multi-encoder transformer architecture with encoders is presented in Fig. 4.

For the proposed concept-based model of this paper, the multi-encoder transformer architecture is designed by adding two encoders, as a special case from the proposed multi-encoder transformer architecture. The first encoder $Enc_1$ includes a self-attention module, that takes its input $Q,K and V$ from the input concept vectors $X_C$, followed by FF network. While the second encoder $Enc_2$ takes its input $Q,K$ and $V$ from the input image’s features $X_F$ and, it is also followed by the FF network.

5 $$\begin{aligned} X_C^n= & {} Enc_1^n (X_C^{(n-1)}) \end{aligned}$$

6 $$\begin{aligned} X_F^n= & {} Enc_2^n (X_F^{(n-1)}) \end{aligned}$$

With the purpose of utilizing the concept information that is represented by the new encoder, a new encoder-decoder attention module is added in the decoder for attending to the output of the new concept encoder. So, each block of the decoder $$Dec^n$$ in the proposed architecture starts with a self-attention module that takes its inputs $$Q,K$$ and $$V$$ from the output captions embedding $$Y$$. Then, the first encoder-decoder attention layer takes its $$K$$ and $$V$$ from the output corresponding to the last block of the first encoder. While the second encoder-decoder attention layer takes its $$K$$ and $$V$$ from the output corresponding to the last block of the second encoder. Finally, the decoder ends with a FF network to transform the attended features. The two encoders and the decoder consist of $$N_x $$identical layers.

7 $$\begin{aligned} Y^n=Dec^n (X_C^N,X_F^N,Y^{(n-1)}) \end{aligned}$$

A vector of floats is produced as an output by the decoder stack. Then, a linear layer takes the vector that is created by the decoder stack and projects it onto a bigger vector, that is referred to as a logit vector. Afterwards, the resulting scores are subsequently turned into probabilities via the Softmax layer.

In comparison with the multi-encoder transformer architecture of^37^, their approach includes two encoders for the source statement and the machine translation output. The decoder includes three Enc-Dec-Atten layers. The first Enc-Dec-Atten layer takes the $K$ and $V$ from Encoder-1 output, i.e., the source sentence, and the $Q$ from the masked MHA layer. The second Enc-Dec-Atten layer takes the $K$ and $V$ from Encoder-1 output and the $Q$ from the Encoder-2, i.e., the output of the machine translation. The third Enc-Dec-Atten layer represents the dependency between Enc-Dec-Atten layer-1 and Enc-Dec-Atten layer-2. To summarize, their approach includes three Enc-Dec-Atten layers for the two encoders.

The proposed multi-encoder transformer architecture (META) of this paper is aimed to be more adequate with the problem being considered in this research, i.e., the image captioning, it is supposed to be simpler and of reduced computational cost relative to^37^.

### Proposed concept-based model for image captioning using multi-encoder transformer architecture (CM-META)

As explained earlier, the research objective of this paper is to enhance the predicted caption of images by employing two feature vectors. The first is the concept vector and the second is the CLIP image feature vector. Therefore, in this paper, the proposed concept-based model for image captioning using a multi-encoder transformer architecture (CM-META) is designed by adding two encoders, as a special case from the proposed multi-encoder transformer architecture. Hence, two Enc-Dec-Atten layers are added to the decoder. The first encoder encodes the image features while the new encoder is added for attending to the concept vectors.

**Figure 5:**
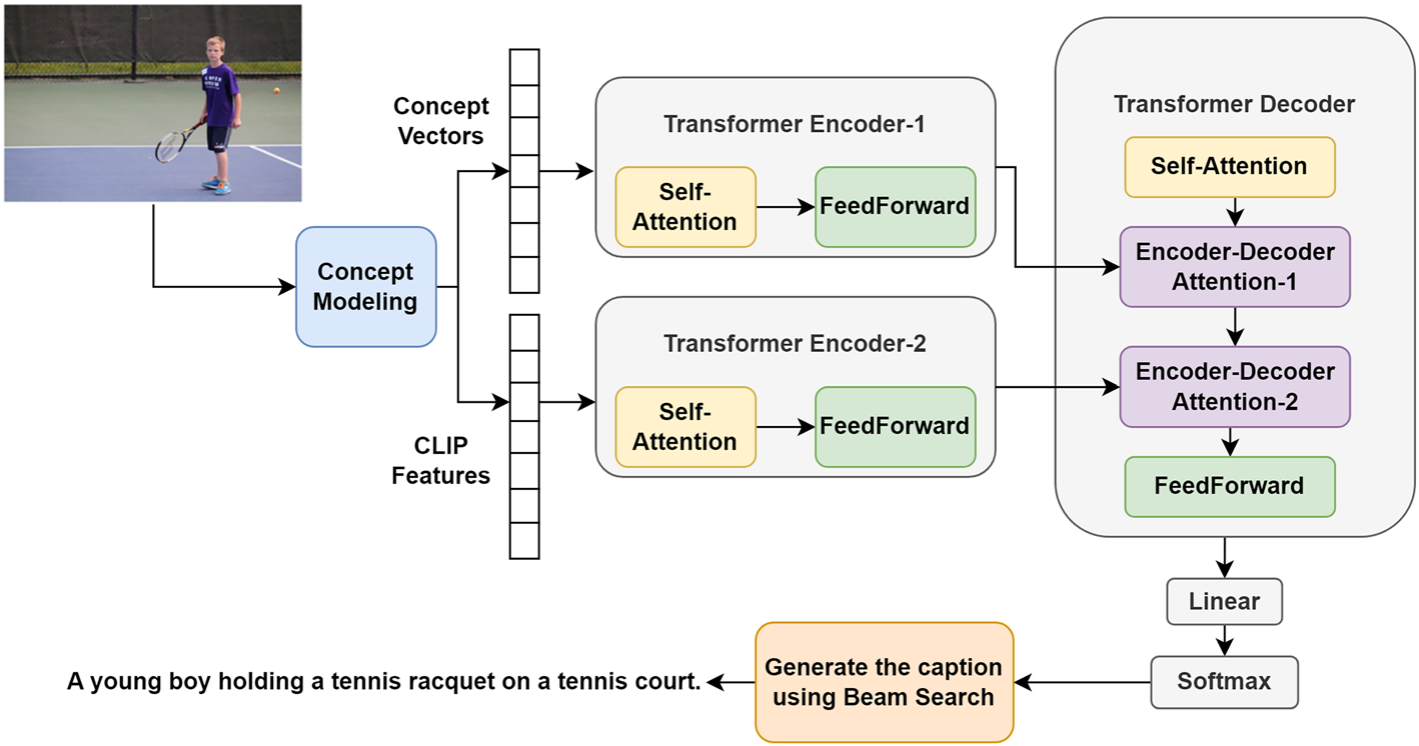
The proposed concept-based model for image captioning using multi-encoder transformer architecture (CM-META) at the inference stage.

Figure 5 presents the proposed concept-based model for image captioning using multi-encoder transformer architecture (CM-META). The proposed model starts by using the concept modeling technique to generate two basic vectors: the first vector represents CLIP image’s embeddings/features; and the second vector is the concept vector which is formulated to capture the image’s semantic information. After that, transformer encoder-1 is used for encoding the concept vectors through two layers namely; self-attention layer and FF-Network. Transformer encoder-2 is utilized for encoding the CLIP image embeddings through the same layers of the first encoder. Afterwards, the partial captions embeddings are fed to a masked multi-head attention (self-attention) layer of the transformer decoder. Then, the first encoder output is fed to the first Enc-Dec-Atten layer of the decoder and the second encoder output is fed to the second Enc-Dec-Atten layer of the decoder. The result after utilizing the transformer is the output words probabilities. These probabilities are then used by beam search^45^ to be converted into words.

## Experimental work and results

### Dataset and evaluation metrics

Microsoft COCO 2017 dataset^12^ and Flickr30k^13^ are used in the performance evaluation of the proposed models. For MSCOCO, there are a minimum of five captions per image in the 123,287-image dataset. Karpathy split^46^ is used to split the MSCOCO^12^ images into 10000 images as validation and test data, each with 5000 images, and the remaining images as training data. For Flickr30K, there are 31,783 images each with 5 captions. Flick30K dataset split assigns 29,000 for train, 1000 for validation and 1000 for test. BLEU^14^, CIDEr^15^, ROUGE^16^, METEOR^17^, and SPICE^18^ metrics are utilized for measuring the predicted captions quality. In addition, training time and GPU type are added to the comparison.

BLEU^14^ metric is utilized to evaluate the quality of text obtained by the machines. The scores for each individual text segment have been obtained through comparing that section to collection of reference texts and analyzing the results. Another metric that evaluates the quality of text summaries is called ROUGE^16^. It does this through comparison of the word sequences, the word pairings, and n-gram to collection of human-generated reference text. METEOR^17^ is used to compare the word segments with reference text. Additionally, phrase roots and word synonyms are taken into account while matching. A greater association may be made via METEOR at the phrase or segment level. CIDEr^15^ is a consensus measure that evaluates picture descriptions automatically. Using TF-IDF, CIDEr obtains human acceptance. On the other hand, the SPICE^18^ metric is utilized for evaluating captions that rely on semantic ideas. It operates on a semantic scene graph. From the captions of the images, this graph may be used to obtain information about various items, their properties, and the connections between them.

### Implementation details

For the purpose of pre-processing the caption texts, the standards are followed by transforming the captions to lower-case, stripping punctuation symbols, and splitting on white spaces. The resulting vocabulary length after considering only words which exist at least five times is 9583, including $<\text {start}>$ and $<\text {end}>$ tokens. The number of concepts resulting from employing the concept modeling technique on MSCOCO data is 81. The embedding dimension is 512 and the transformer parameters are 8 heads and 3 layers. Adam optimizer^47^ is utilized with learning rate equals $2e^{-5}$. The proposed concept-based models have been trained for minimizing cross-entropy loss. Training of the models run for 20 epochs on a single Nvidia GTX1080 GPU. Beam search is used in the testing phase with a beam size of 2. For the Flickr30K dataset, we have used the same parameters in obtaining the performance results.

### Quantitative analysis

The quantitative results have been obtained using the MSCOCO caption evaluation code, which includes the standard metrics BLEU^14^, CIDEr^15^, ROUGE^16^, METEOR^17^, and SPICE^18^. Table 3 presents a comparison between the proposed models relative to the state-of-the-art topic-based image captioning models using BLEU (B@1, B@2, B@3, B@4)^14^, CIDEr^15^, ROUGE^16^, METEOR^17^, and SPICE^18^ metrics on the MSCOCO dataset. In addition, training time and GPU type are added to the table. Six different topic-based approaches are included in the table. The approaches had topic modeling as their main contribution and used different topic, image, and language models. In addition, NumCap proposed by Abdussalam et al.^48^ has been added to the comparison table. The NumCap approach generates multiple output captions instead of resulting only one caption like all captioning models. Abdussalam et al.^48^ assigned a number to each ground truth caption then they use a pair of the image and its number as input to their captioning model.

It is clear in Table 3 that the results of the proposed CM-LSTM model surpassed the results of the approaches^7,22,23,30,48^ and achieved the minimum training time. It means that using only the concept modeling technique with its CLIP embeddings in an LSTM-based language model can enhance the results even without using attention or transformer. In addition, the results of the proposed CM-META surpassed the results of all related works. Table 4 includes a comparison between the proposed models (CM-LSTM and CM-META) and the related work approaches^48^ and^22^ which include the Flickr30K results in their comparisons. It is clear from the table that the proposed models achieved better performance with respect to the standard evaluation metrics in comparison with the related works. Which ensures the ability of the concept modeling technique to produce better captions’ quality than topic modeling by depicting the image contexts very well.

**Table 3:** Comparisons of the proposed concept-based models (CM-LSTM, CM-META) with respect to the state-of-the-art topic-based approaches for image captioning on the MSCOCO dataset.

Captioning Model	B@1 $\uparrow$	B@2 $\uparrow$	B@3 $\uparrow$	B@4 $\uparrow$	METEOR $\uparrow$	CIDEr $\uparrow$	ROUGE $\uparrow$	SPICE $\uparrow$	Training Time $\downarrow$
NumCap^48^	66.9	49.4	36.5	27.3	24.1	85.3	50.7	17.0	–
Topic-based captioning^7^	67.6	49.4	34.8	24.3	22.7	80.8	49.3	–	6h (GTX1080)
Topic-sensitive^30^	72.1	53.4	40.6	24.1	20.1	67.3	–	–	–
Show and tell more^22^	72.3	54.1	39.2	28.9	23	90.3	52.1	–	–
What Topics Do Images Say^23^	73.3	56.0	41.1	30.1	25.2	98.6	53.4	–	–
Topic-oriented (NeuralTalk2-T-oe)^28^	73.9	57.2	43.2	32.6	26.1	103.8	54.4	–	–
Topic-guided Attention (VA)^8^	75.2	56.16	41.4	30.4	27.0	109.2	**58.1**	–	8h (Single NVIDIA TITAN X GPU)
Proposed CM-LSTM	73.7	56.8	41.9	30.5	25.8	99.1	53.9	19.3	**4h** (GTX1080)
Proposed CM-META	**75.8**	**59.6**	**45.3**	**34.1**	**27.4**	**110.1**	56.0	**20.6**	**5h** (GTX1080)

**Table 4:** Comparisons of the proposed CM-LSTM and CM-META approaches with respect to the state-of-the-art topic-based approaches on the Flickr30k dataset.

Captioning Model	B@1 $\uparrow$	B@2 $\uparrow$	B@3 $\uparrow$	B@4 $\uparrow$
NumCap^48^	58.9	40.5	27.3	18.2
Show and tell more^22^	69.1	47.2	31.2	20.8
Proposed CM-LSTM	68.4	48.2	32.9	24.8
Proposed CM-META	**70.4**	**51.2**	**34.5**	**25.9**

**Figure 6:**
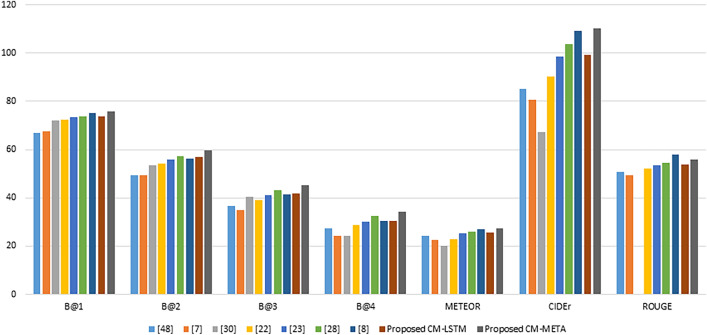
Comparison of the proposed concept-based models (CM-LSTM, CM-META) with the topic-based models with respect to the standard evaluation metrics on the MSCOCO dataset.

Figure 6 presents a bar chart comparison between the proposed models (CM-LSTM, CM-META) and the state-of-the-art topic-based approaches for image captioning with respect to the standard metrics. The proposed CM-META model achieved the best results according to the evaluation metrics in comparison with the topic-based approaches. Topic-guided Attention approach (VA)^8^ achieved higher ROUGE value in comparison with the proposed CM-META model. However, the Topic-guided Attention approach (VA)^8^ requires longer training time and used two types of attention (semantic and visual attentions). The proposed CM-META required less computational cost related to all state-of-the-art topic-based techniques, according to Table 3. Which means that utilizing the concept modeling technique instead of the topic modeling, especially with the multi-encoder transformer architecture, is capable of enhancing the image captioning performance. This enhancement is due to the rich semantics that result from utilizing the concept modeling technique, which are represented in the concept vectors. In addition, the proposed models were able to make use of this semantic information while predicting the captions and reduced the computational complexity.

**Figure 7:**
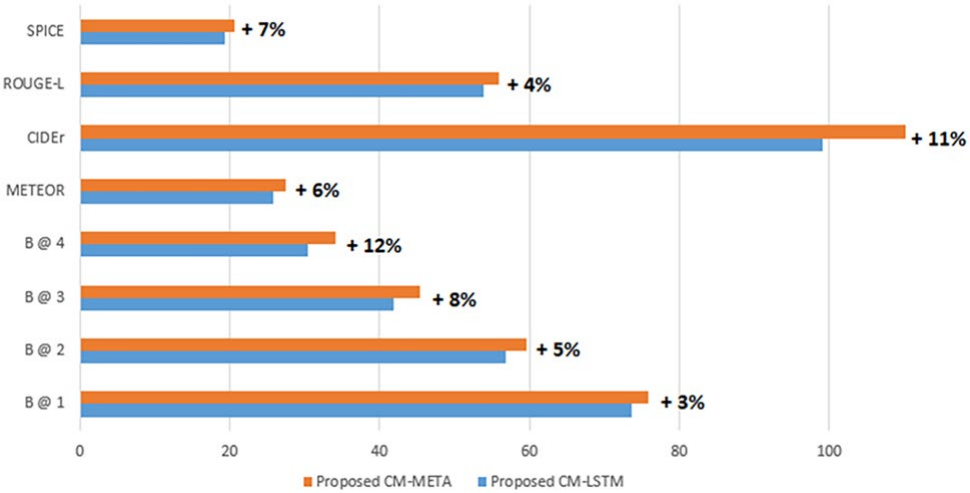
A bar chart comparison between the proposed CM-LSTM model and the proposed CM-META model in terms of the standard metrics on the MSCOCO dataset.

Figure 7 presents a bar chart comparison of the proposed CM-LSTM model relative to the proposed CM-META model based on the evaluation metrics. The proposed CM-META model enhanced the results with 12% in BLEU-4, 6% in METEOR, 11% in CIDEr, and 7% in SPICE scores, as represented in Fig. 7. The proposed CM-META model improved the image captions by utilizing the novel concept vector in a transformer-based language model. The new added encoder in the transformer encoded the concepts information which are then utilized by an Enc-Dec-Atten layer in the transformer decoder to represent the image semantics in the produced caption.

Finally, it is clearly seen from the above conducted comprehensive comparative study that the two proposed image captioning models were able to achieve improved image captions with respect to the state-of-the-art image captioning techniques. Further, this enhancement has been achieved by the proposed concept-based models for image captioning at a reduced computational cost relative to all state-of-the-art techniques found in the literature.

**Table 5:** Some caption results on the MSCOCO test data using the proposed concept-based models (CM-LSTM, CM-META).

Input image	Ground truth caption	Generated caption using proposed CM-LSTM	Generated caption using proposed CM-META
	A cat sits on the top of a computer desk.	A cat laying on a desk next to a computer.	A black and white cat laying on top of a computer keyboard.
	A bear is walking away from a car.	A black bear standing on a paved road.	A large black bear walking across a road.
	A man and woman standing on a tennis court.	A man and a woman standing next to each other.	A man and a woman playing tennis in a tennis court.
	A small bird patiently sits on a tree branch.	A small bird sitting on top of a tree.	A black and white bird sitting on a tree branch.
	A few people are getting ready to ski.	A group of people riding skis on top of snow covered ground.	A group of people riding skis down a snow covered slope.
	A unique fire hydrant is pictured in this image.	A fire hydrant sitting in the middle of a road.	An old fire hydrant sitting on the side of a road.
	A small dog sitting on top of a grass yard.	A dog is laying on the grass outside.	A dog sitting on the grass outside of a house.

### Qualitative analysis

In order to qualitatively analyze the proposed models’ performance, some of the generated captions by the proposed concept-based models are added in Table 5. The images included in Table 5 belongs to the MSCOCO dataset^12^ (test split). ). The dataset is publicly available (MSCOCO Download,“https://cocodataset.org/#download”) and the terms of use for this dataset are available at their official website (MSCOCO Terms of use, “https://cocodataset.org/#termsofuse). In addition to including the generated captions of the proposed models, the table also includes the corresponding ground truth human-generated captions for the MSCOCO test data. As presented in Table 5, the proposed models can depict the images quite accurately. The proposed models can generate better captions while concentrating on the concept of the images. That can be seen in Table 5 when adding “black” to describe the “bear” in the second image. For the fourth image, the proposed CM-META model was able to determine that the “bird” is “black and white” which is more accurate than the ground truth. In addition, adding the “road” when describing the sixth image of the fire hydrant was more expressive, however, the proposed CM-META model was able to determine that the fire hydrant is old and it was on the side of the road not the middle. The proposed CM-LSTM model was able to accurately caption the seventh image but the proposed CM-META model produced better caption by determining that the “dog” is “outside of a house” by considering the background of the image.

## Conclusion and future work

Novel concept-based models were proposed in this paper for image captioning task. Concept modeling technique was utilized for extracting more valuable information from the images which are represented in the concept vectors. The proposed models explain how to incorporate the concept vectors as extra guiding input in two different language models. First, concept-based model was proposed for image captioning using LSTM. Then, for the purpose of producing more expressive captions at lower computational complexity, a new proposed multi-encoder transformer architecture is used in the second proposed concept-based model. It was proposed to modify the traditional transformer by having multi-encoder architecture for representing the novel concept vectors in the captioning process. The proposed concept-based models have been compared quantitatively with state-of-the-art approaches. The proposed models enhanced the results, regarding the standard metrics, in comparison to the state-of-the-art approaches. Further, this improvement has been accomplished at a reduced computational complexity. The proposed concept-based models were compared qualitatively using examples of the produced captions by the proposed models to the ground truth captions and showed more accurate descriptions. Finally, it is clear that utilizing the concept modeling technique in the image captioning task improved the image captioning performance. Moreover, utilizing the multi-encoder transformer architecture achieves a significant improvement in the image captioning field in terms of the evaluation metrics as well as the computational cost.

In the future, an optimization technique can be used for selecting the number of transformer heads and layers in order to achieve better results. The proposed concept-based model utilizing META may be used in video captioning task. In addition, the proposed multi-encoder transformer architecture can be used for tasks other than the image captioning task.

## Data Availability

The dataset used in the analysis of this study is available in COCO website: https://cocodataset.org/. The terms of use for this dataset are included in this link: https://cocodataset.org/#termsofuse.
